# Clinical characteristics and prognosis of COVID-19- associated invasive pulmonary aspergillosis in critically patients: a single-center study

**DOI:** 10.3389/fcimb.2025.1522217

**Published:** 2025-04-22

**Authors:** Shuang Xiao, Jie Xu, Han Xiao, Yonggang Li, Xu Chen, Li Chen, Weifeng Zhao

**Affiliations:** ^1^ Department of Infectious Diseases, The First Affiliated Hospital of Soochow University, Suzhou, China; ^2^ Center of Clinical Laboratory, The First Affiliated Hospital of Soochow University, Suzhou, China; ^3^ Department of Infectious Disease, Qingdao Municipal Hospital, Qingdao, China; ^4^ Department of Radiology, The First Affiliated Hospital of Soochow University, Suzhou, China

**Keywords:** COVID-19, invasive pulmonary aspergillosis, mortality, influencing factors, clinical characteristics, microbiological characteristics

## Abstract

**Objective:**

A single-center retrospective study was conducted according to the latest diagnostic criteria of the European Consortium for Mycology in Medicine/International Society for Human and Animal Mycoses (ECMM/ISHAM) Consensus, which describes the clinical characteristics, factors influencing and prognosis of a group of patients with COVID-19 (Omicron variant) combined with invasive pulmonary mycoses with onset of disease at the end of 2022.

**Methods:**

This study retrospectively analyzed data related to 58 hospitalized patients with severe pneumonia due to COVID-19 infection admitted to the ICU of critical care medicine, respiratory ICU, and ICU of the Department of Infections at the First Affiliated Hospital of Soochow University from December 1, 2022, to January 31, 2023. CAPA was defined according to the ECMM/ISHAM consensus criteria. Our study compared the clinical and microbiological characteristics and associated risk factors of fungal infections and pulmonary fungal infections and performed univariate and multivariate analyses of factors associated with mortality in patients with COVID-19-Associated Pulmonary Aspergillosis (CAPA).

**Results:**

17 (29.3%) of the 58 critically ill patients were diagnosed with CAPA, of which 10 (58.82%) patients were Probable CAPA and 7 (41.18%) patients were Possible CAPA. Among this *Aspergillus* strains, *Aspergillus fumigatus* strains were found in 13 cases (76.47%) and *Aspergillus niger* strains in 4 cases (23.53%). 7 (41.18%) patients had concomitant bacterial fungal infections with a mortality rate of 57.14% (4/7), of which *Acinetobacter baumannii* was the most common pathogen. Among the patients with CAPA, galactomannan assay of bronchoalveolar lavage fluid (BALF) was performed in 5 patients with a 100% (5/5) positivity rate, and two or more serum galactomannan (GM) assays were performed in 17 patients, with a probability of favorable results in both cases of 41.2% (7/17). The 60-day mortality rate in patients with CAPA was 52.9% (9/17), whereas the non-CAPA patients had a 60-day mortality rate of 24.4% (10/41), which was statistically different (P = 0.035). Diabetes mellitus (P = 0.018, OR: 5.040 (95% CI: 1.314-19.337)), renal insufficiency (P=0.002, OR: 11.259 (95% CI: 2.480-51.111)), chronic obstructive pulmonary disease (COPD) (P = 0.003, OR: 6.939 (95% CI: 1.963-24.531)), elevated interleukin-6 (IL-6) (P = 0.022, OR: 4.160 (95% CI: 1.226~14.113)), mechanical ventilation (P = 0.002, OR: 8.100 (95%CI: 2.132~30.777)), increased duration of steroids use (P = 0.022, OR: 1.071 (95%CI: 1.010~1.135)), increased cumulative dose of steroids use ((P < 0.001, OR: 1.012 (95%CI: 1.009~1.015)), use of tocilizumab (P = 0.020, OR: 11.480 (95%CI: 2.480~51.111)), and increased length of hospitalization in ICU (P = 0.021, OR: 1.038 (95% CI: 1.006 to 1.071)), and increase in the type of antibiotics used (P = 0.002, OR: 1.603 (95% CI: 1.181 to 2.176)) were the risk factors for the occurrence of fungal infections, whereas the use of steroids or not, the use of baricitinib or not, and hypertension did not have a significant effect on the occurrence of fungal infections (P > 0.05). Patients with CAPA had a higher mortality rate, and their hospitalization was prolonged compared to non-CAPA patients. The all-cause mortality rate for patients with CAPA was 52.9%. We also performed univariate and multivariate analyses of potential factors associated with mortality, including the use of mechanical ventilation (P = 0.040 OR: 10.500, (95% CI: 1.115 to 98.914)), advanced age (P = 0.043 OR: 1.212, (95% CI: 1.006 to 1.460)), and a significantly higher CRP level (P = 0.042 OR: 1.043, (95% CI: 1.002~1.078)) had a worse prognosis. Steroids use, gender, and diabetes mellitus were not associated with patient death (P > 0.05).

## 1 Introduction

At the end of 2022, the global COVID-19 outbreak resumed, with the Omicron variant becoming dominant. This variant has numerous mutations that boost its ability to evade humoral immunity and increase transmissibility (Rana et al., 2022). Although Omicron infections are generally less severe, the extreme transmissibility led to a surge in cases and an increase in critically ill patients (Brüssow, 2022; Wu Y. et al., 2022). COVID-19 patients in intensive care units (ICUs) face higher risks of co-infections and mortality (Binkhamis et al., 2023). Pulmonary aspergillosis, an opportunistic infection linked to mechanical ventilation, predominantly affects ICU patients with chemotherapy, transplants, or immunosuppression (Thompson et al., 2020). During COVID-19 epidemics, the virus was found to damage alveolar epithelial and tissue endothelial cells, disrupting lung barriers (Short et al., 2016). Combined with prolonged hospital stays, steroid and immunomodulator use, and the need for invasive mechanical ventilation in most patients with acute respiratory distress syndrome, this makes critically ill COVID-19 patients more prone to bacterial and opportunistic fungal lung infections (*Aspergillus*, *Candida*, *Cryptococcus*, and *Trichinella*), with *Aspergillus* being the most common (Chiurlo et al., 2021). Some studies have reported that the prevalence of CAPA in patients with severe COVID-19 ranges from 2.4% to 34.3% (Kariyawasam et al., 2022). The prognosis for severe CAPA is relatively poor, characterized by high morbidity and mortality rates that range from 22.2% to 71.4% (Kariyawasam et al., 2022; Lamoth et al., 2021; Pasquier et al., 2021). This situation poses a significant threat to public health. Since the beginning of the COVID-19 pandemic, many studies on CAPA have been reported, and the prevalence of CAPA in COVID-19 ICU patients reported in different studies varies widely, which may be related to inconsistent surveillance methods and diagnostic criteria (Chong et al., 2022). In 2020, the European Confederation of Medical Mycology/International Society for Human and Animal Mycoses consensus developed relevant diagnostic criteria and disposition consensus for CAPA. The diagnosis of *Aspergillosis* was categorized as Proven CAPA (histopathological confirmation or *Aspergillus* positive in sterile samples), Probable CAPA (host factors + clinical deterioration + mycological evidence e.g., BALF GM ≥1.0), and Possible CAPA(clinical suspicion with insufficient mycological evidence/non-specific findings), and the relevant host factors that predispose to aspergillosis were defined (Asperges et al., 2024). While this consensus aids in diagnosing and treating CAPA, it remains a clinical challenge (Skóra et al., 2023).

There are few studies on the incidence, prognosis, and associated risk factors of CAPA in the Omicron variant. This retrospective study analyzed CAPA incidence in the Omicron variant, identified risk factors, and assessed their relationship with patient prognosis. The findings may guide future viral pneumonia treatment, reduce secondary pulmonary mycoses, and improve severe COVID-19 pneumonia survival rates.

## 2 Materials and methods

### 2.1 Study population and inclusion-exclusion criteria

We conducted a single-center retrospective observational cohort study of patients admitted to three ICUs (Intensive Care Medicine ICU, Respiratory ICU, and Infectious Disease ICU) of the First Affiliated Hospital of Soochow University between December 1, 2022 and January 31, 2023. No personally identifiable information about participants was accessed during data collection. Inclusion criteria were 1. age ≥18 years; 2. positive SARS-CoV-2 polymerase chain reaction (PCR) test on nasopharyngeal swabs or respiratory samples; and 3. patients diagnosed with COVID-19 severe pneumonia. Exclusion criteria were: 1. patients who died or were automatically discharged within 48 hours of admission; 2. patients who lacked sufficient clinical data; and 3. patients who contracted COVID-19 pneumonia during hospitalization (**Table 1**).

**Table 1:** Criteria for inclusion and exclusion.

Category	Criteria
Inclusion	1. Adults ≥18 years
	2. Confirmed SARS-CoV-2 infection (PCR-positive respiratory sample)
	3. Diagnosis of severe COVID-19 pneumonia
Exclusion	1. Death or self-discharge within 48 hours of admission
	2. Insufficient clinical documentation
	3. Hospital-acquired COVID-19 pneumonia

### 2.2 Data collection

CAPA in this study was defined according to the consensus criteria for research and clinical guidance published by ECMM/ISHAM 2020. These criteria defined CAPA cases as Proven CAPA, Probable CAPA, and Possible CAPA. This paper collects Probable CAPA and Possible CAPA.

Clinical data, as well as pathogenetic data, were collected from COVID-19 patients, including demographic characteristics, length of hospitalization, inflammatory biomarkers, fungal isolates, infectious agents (*Klebsiella pneumoniae*, *Pseudomonas aeruginosa*, *Escherichia coli*, *Enterobacter cloacae*), underlying diseases [Diabetes mellitus, Hypertension, Hepatic disease, Cardiovascular disease, Chronic kidney disease (CKD), Chronic obstructive pulmonary diseases (COPD), personal history of malignancy, whether mechanically ventilated or not, duration of mechanical ventilation]. The primary outcome indicator was to assess the incidence of proposed or Possible CAPA. Secondary outcome indicators identified CAPA risk factors and assessed the association between relevant risk factors and 60-day mortality. Detailed information on the above can be found in **Table 2**.

**Table 2:** Univariate analysis of risk factors for the occurrence of CAPA.

Characteristics	CAPA (n=17)	NO CAPA (n=41)	Total (n=58)	*P* value
Demographics				
Male [Cases (%)]	12 (70.59)	32 (78.05)	44(75.86)	0.071
Age, median (IQRs)	76.0 (63.0,84.0)	78.0 (71.0,84.0)		0.991
Underlying diseases				
Diabetes [Cases (%)]	7 (41.18)	5 (12.29)	12 (20.69)	**0.034**
Hypertension [Cases (%)]	9 (52.94)	18 (43.90)	27 (46.55)	0.530
Cerebral infarction [Cases (%)]	3 (17.65)	5 (12.29)	8 (13.79)	0.681
Hematencephalon [Cases (%)]	1 (5.88)	2 (4.88)	3 (51.72)	1.000
Cardiovascular disease [Cases (%)]	2 (11.76)	6 (14.63)	8 (13.79)	1.000
Hepatic disease [Cases (%)]	2 (11.76)	5 (12.19)	7 (12.07)	1.000
Renal insufficiency [Cases (%)]	8 (47.06)	3 (7.32)	11 (18.97)	**0.001**
COPD [Cases (%)]	10 (58.82)	7 (17.07)	17 (29.31)	**0.003**
Cancer [Cases (%)]	8 (47.06)	10 (24.39)	18 (31.03)	0.089
Laboratory indicators				
High CRP (IQRs)	135.24 (88.34,219.55)	32 (45.1)		0.274
Elevated IL-6 [Cases (%)]	12 (70.59)	15 (36.59)	27(46.55)	**0.020**
Leucocyte (IQRs)	8.03 (3.76-11.26)	6.5 5(4.47-9.34)	6.68 (3.91-10.39)	0.447
Methods of treatment				
Invasive mechanical ventilation [Cases (%)]	9 (52.94)	5 (12.20)	14 (24.14)	**0.001**
Eat autonomously [Cases (%)]	12 (70.59)	26 (63.41)	38 (65.52)	0.743
Steroids [Cases (%)]	15 (88.24)	32 (78.05)	47 (81.03)	0.789
Steroids Use time, median (IQRs)	15 (10,27)	10 (6.25,16.75)		**0.026**
Cumulative steroid [mg]	89.00(75.00,101.75)	54.00 (37.50,89.00)		**<0.001**
Tolizumab [Cases (%)]	8 (47.06)	3 (7.32)	11 (18.97)	**0.001**
Baricitinib [Cases (%)]	9 (52.94)	13 (31.71)	22 (37.93)	0.153
Length of ICU stay, median (IQRs)	45(31,62)	30 (20,41)		**0.049**
Types of antibiotics (IQRs)	5(3.5-6.0)	2 (1.0-4.0)	3 (2.0-5.0)	**<0.001**
In-hospital mortality [Cases (%)]	9(52.94)	10 (24.39)	19 (32.76)	**0.035**

Statistically significant intergroup differences (*P*<0.05) are highlighted in boldface type.

### 2.3 Statistical methods

Descriptive statistics were used to assess the baseline characteristics of the entire cohort; the variables involved in this study showed a skewed distribution, and the information of the skewed distribution was expressed as median (M) and interquartile ranges (IQR). Quantitative variables were compared using Student’s t-test or Mann-Whitney U-test; we used the Pearson chi-square test or Fisher exact test for qualitative variables. Variables considered clinically relevant and statistically significant in the univariate analysis were included in the multivariate logistic regression analysis. All statistical analyses were performed using IBM SPSS version 22.0 software (IBM et al., USA), and a P value of < 0.05 was considered statistically significant.

## 3 Results

### 3.1 Diagnosis of CAPA and its clinical characteristics

**Figure 1:**
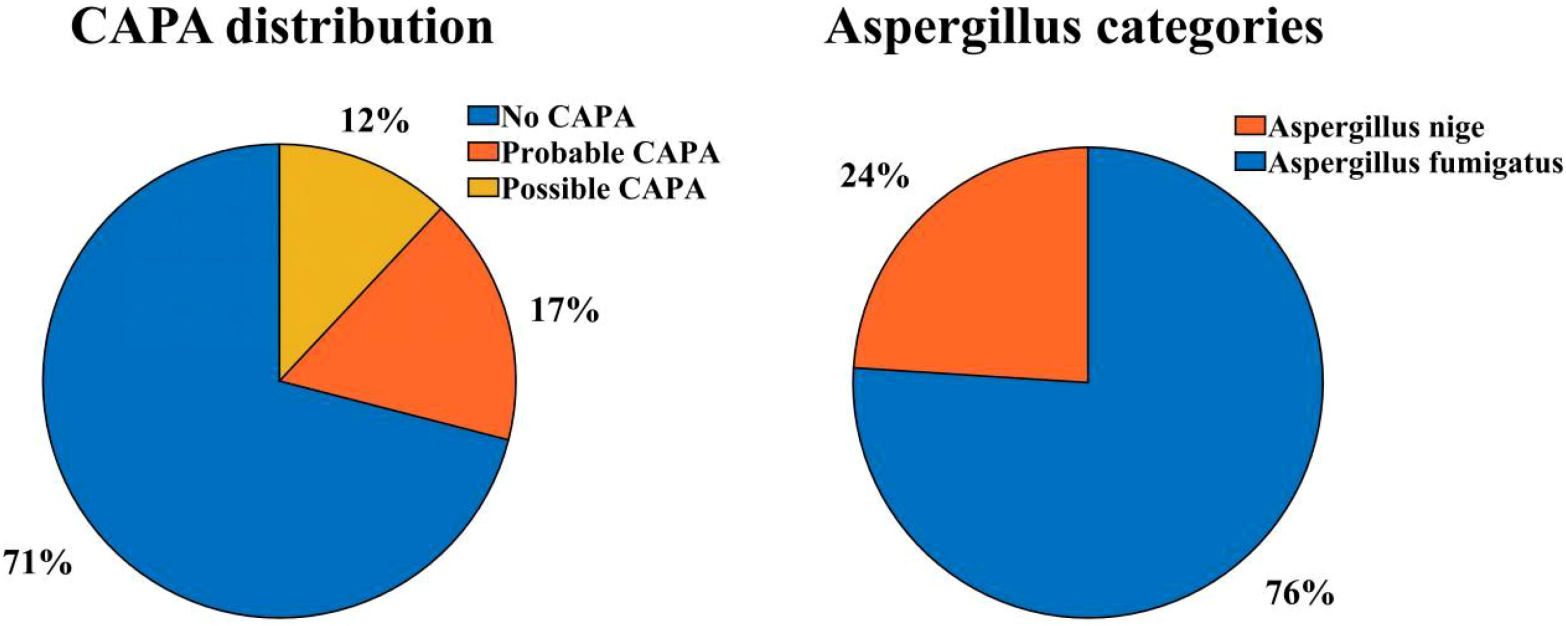
CAPA distribution and aspergillus categories.

From December 1, 2022, to January 31, 2023, a total of 67 patients were diagnosed with severe COVID-19 pneumonia and admitted to the three ICUs of our hospital (Critical Care Medicine ICU, Respiratory ICU, and Infectious Disease ICU), of which 58 patients were enrolled in this study. Among the enrolled critically ill patients with COVID-19, the incidence of CAPA was 29.3% (17/58), 10 (58.82%) patients were diagnosed with Probable CAPA, and 7 (41.18%) patients were diagnosed with Possible CAPA (**Figure 1**).

**Figure 2:**
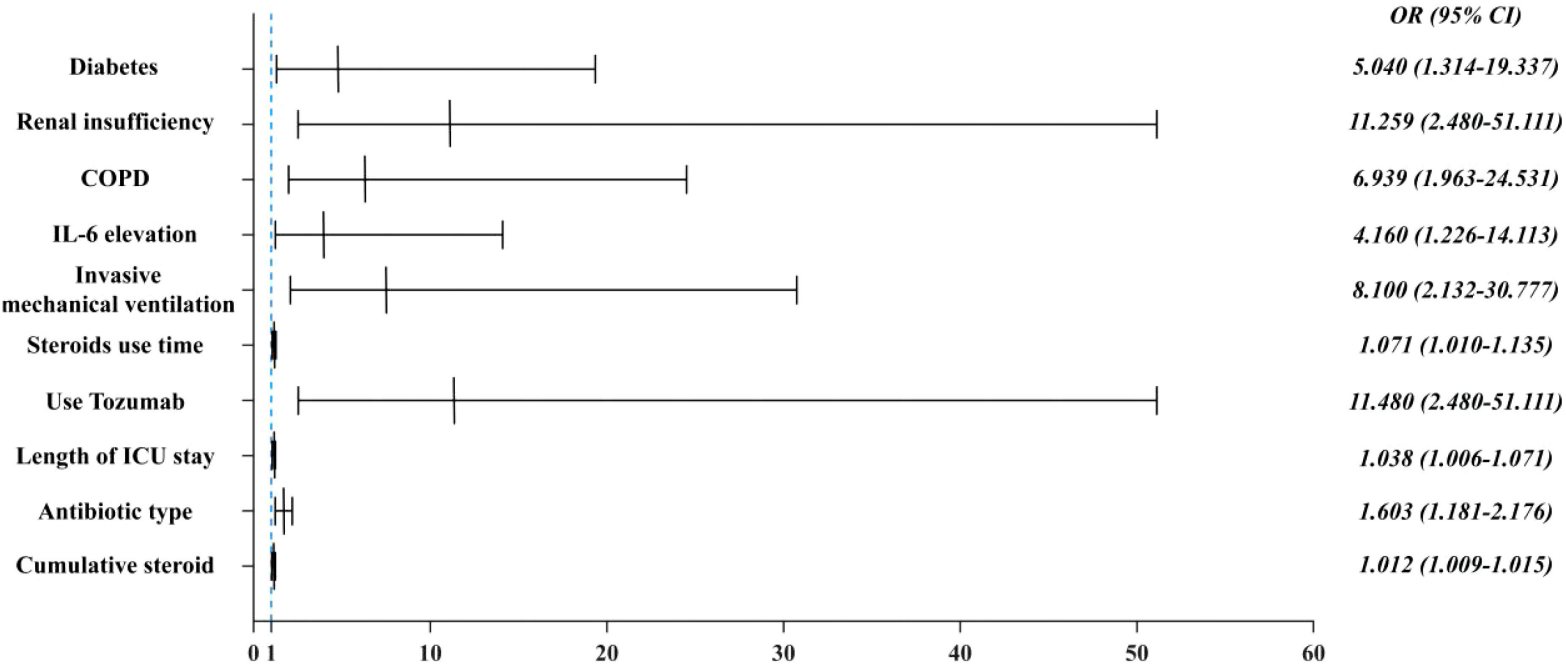
Multifactorial analysis of risk factors for the occurrence of CAPA.

**Figure 3:**
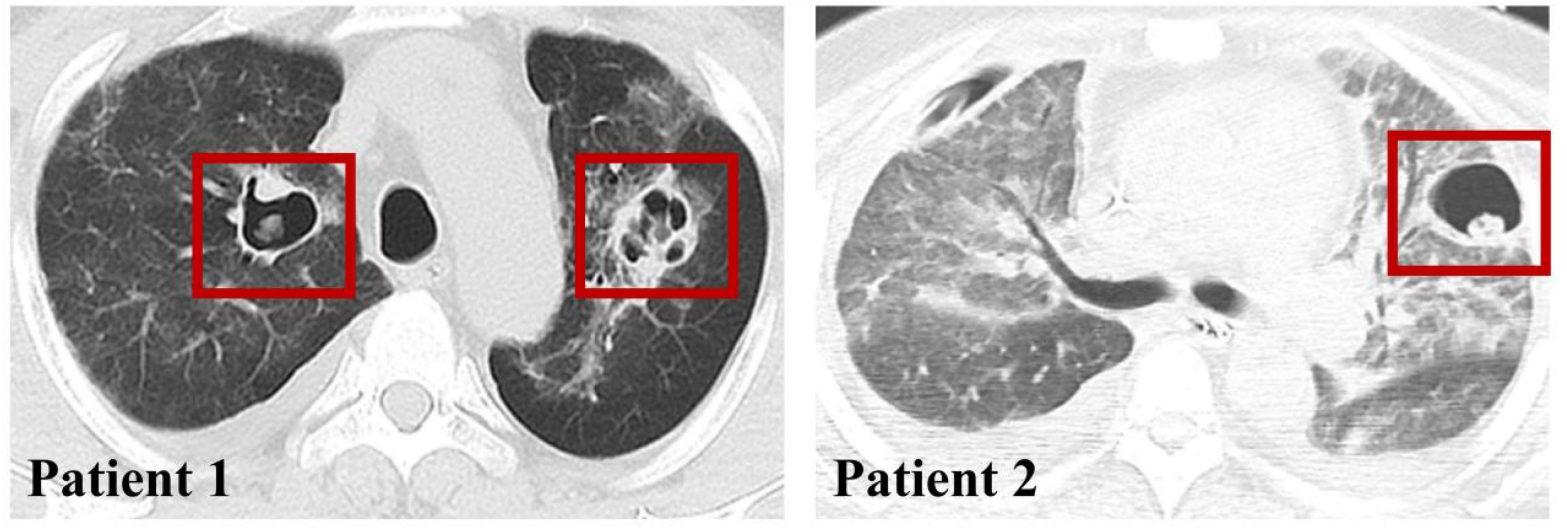
CAPA imaging presentation.

We performed univariate and multifactorial analyses of risk factors that may influence the occurrence of CAPA. We found that elderly male patients accounted for most CAPA and non-CAPA patients. Regarding the patients’ underlying diseases, we found that the combination of diabetes (P=0.018, OR: 5.040 (95% CI: 1.314~19.337)), renal insufficiency (P=0.002, OR: 11.259 (95% CI: 2.480~51.111)), COPD (P = 0.003, OR: 6.939 (95% CI: 1.963~24.531)) was more likely to develop CAPA (**Figure 2**), while comorbidities such as hypertension, cerebral infarction, hematencephalon, hepatic disease, and cancer did not show statistically significant differences in whether CAPA occurred (**Table 2**). Among some inflammatory indicators, elevated IL-6 increased the incidence of CAPA in COVID-19 critically ill patients (P = 0.022, OR: 4.160 (95% CI: 1.226-14.113)) (**Figure 2**). In addition, mechanical ventilation (P = 0.002, OR: 8.100 (95% CI: 2.132~30.777)), the use of tolizumab (P = 0.020, OR: 11.480 (95% CI: 2.480~51.111)), and an increase in length of hospitalization (P = 0.021, OR: 1.038 (95% CI: 1.006~ 1.071)) could all increase the risk of infection in CAPA (**Figure 3**). This study also found that the majority of patients with both CAPA and non-CAPA patients used steroids therapy, with 15 (88.2%) patients with CAPA using steroids within three days of admission and 32 (78.0%) patients with non-CAPA using steroids within three days of admission, and that there was no statistically significant difference between steroids use and non-use of steroids for the occurrence of CAPA, and that As the duration of steroids use increased, the odds of CAPA increased (P < 0.001, OR: 0.043 (95% CI: 0.008~0.221), and the cumulative amount of steroids use increased, the odds of CAPA also increased (P < 0.001, OR: 1.012 (95% CI: 1.009~1.015). At the same time, we also found that the type of antibiotics used before the occurrence of CAPA was higher in CAPA patients than in non-CAPA patients (5 (3.5-6.0) vs. 2 (1.0-4.0)) and that the occurrence of CAPA was also associated with an increase in the type of antibiotics used (P = 0.002, OR: 1.603 (95% CI: 1.181~2.176)) (**Figure 2**).

### 3.2 Pathogenetic and imaging characteristics

In this study, all patients underwent more than two sputum cultures, positive for *Aspergillus*. 5 patients underwent bronchoscopic examination, and the Bronchoalveolar Lavage Fluid (BALF) was retained and cultured to reveal *Aspergillus*. Positive strains included the *Aspergillus fumigatus* strain in 13 cases (76.47%) and the *Aspergillus niger* strain in 4 cases (23.53%). 7 (41.18%) patients had co-infections of bacterial and fungal infections, predominantly gram-negative bacteria, most frequently *Acinetobacter baumannii*, and most bacteria were multiresistant. All patients with CAPA underwent more than two serum Galactomannan (GM) tests, and the probability rate of serum mean GM index > 0.5 was 41.2% (7/17). Considering that the COVID-19 virus can be transmitted by aerosol, to safeguard the safety of medical personnel, only five patients in this study underwent BALF GM test, and the GM index were all ≥ 1.0. All CAPA patients were initiated with antifungal drugs at the time of sputum culture or BALF suggestive of *Aspergillus* positivity, and the most commonly used antifungal drug was voriconazole (9/17, 52.94%), followed by esaconazole (5/17, 29.41%) and amphotericin B (3/17, 17.65%) was also used in some patients. All patients were on broad-spectrum antibiotics prior to the onset of CAPA.

In addition, all patients underwent chest CT, and only 2 patients were seen to have vacuolar infiltrate formation in the lungs (**Figure 3**), while the other portion presented with a diffuse infiltrative presentation of the lungs.

### 3.3 Mortality

**Figure 4:**
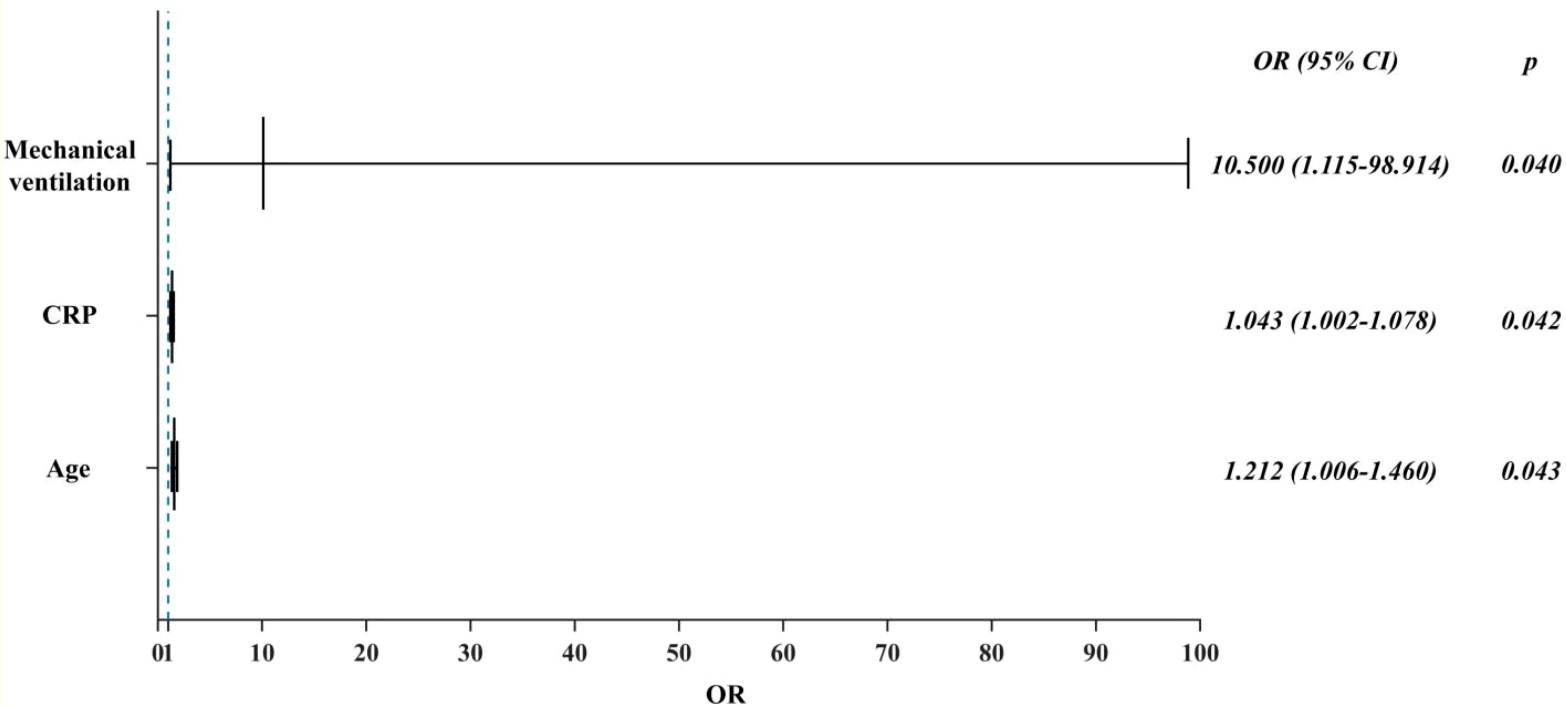
Multifactorial analysis of factors influencing the prognosis of CAPA patients.

As presented in **Table 3**, the 60-day mortality rate was 52.9% (9/17) in CAPA patients and 24.4% (10/41) in non-CAPA patients, with a statistically significant difference in the 60-day mortality rate between the two (P=0.004, OR: 0.287 (95% CI: 0.087-0.942)). We performed a univariate analysis of the 60-day morbidity and mortality rates by various clinical characteristics affecting patients with CAPA. The results indicated that age (P < 0.001), mechanical ventilation (P = 0.015), duration of steroids administration (P < 0.001), and elevated C-reactive protein(CRP) (P < 0.001) had statistically significant differences in whether a patient died or not. However, when these risk factors were included in a multifactorial Logistic regression analysis, the results showed that the use of mechanical ventilation (P = 0.040 OR: 10.500, (95% CI: 1.115-98.914)) (**Figure 4**), advanced age (P = 0.043 OR: 1.212, (95%CI: 1.006-1.460)) and CRP levels were significantly higher (P = 0.042 OR: 1.043, (95% CI: 1.002~1.078)) (**Figure 4**), while gender and duration of steroids use had no significant effect on prognosis (P > 0.05) (**Table 3**).

**Table 3:** Univariate analysis of risk factors affecting the prognosis of CAPA patients.

	Dead (n=9)	Alive (n=8)	Total (n=17)	*P* value
Male [Cases (%)]	9 (100.00)	3 (37.50)	12 (70.59)	0.090
Age, median (IQRs)	76.00 (65.00,85.00)	68.50 (50.25,84.00)	76.0 (63.0,84.0)	**<0.001**
Invasive mechanical ventilation [Cases (%)]	7 (77.78)	2 (25.00)	10 (58.82)	**0.015**
Hypertension [Cases (%)]	5(55.56)	4 (50.00)	9 (52.94)	1.000
Diabetes[Cases (%)]	5 (55.56)	2 (25.00)	7 (41.18)	0.335
Hematencephalon[Cases (%)]	1 (11.11)	0 (0.00)	2 (25.00)	1.000
COPD [Cases (%)]	7 (77.78)	3 (37.50)	10 (58.82)	0.153
Renal insufficiency [Cases (%)]	3 (33.33)	1 (12.50)	4 (23.53)	0.576
Steroids [Cases (%)]	9 (100.00)	6 (75.00)	15 (88.24)	0.206
Steroids Use time, median (IQRs)	19.00 (12.00,31.00)	12.50 (7.50, 22.75)	15 (10,27)	**<0.001**
Cumulative steroid [mg]	75.00 (74.07,118.13)	95.44 (75.19,122.25)		0.862
Tozumab [Cases (%)]	5 (55.56)	3 (37.50)	8 (47.06)	0.637
CRP, median (IQRs)	192.00 (119.54,219.55)	88.34 (36.33,134.00)	135.24 (88.34,219.55)	**<0.001**
Elevated IL-6 [Cases (%)]	7 (77.78)	5 (62.50)	12 (70.59)	0.620
Elevated PCT [Cases (%)]	9 (100.00)	6 (75.00)	15 (88.24)	0.206
Leucocytemedian (IQRs)	12.52 (8.03,11.69)	10.38 (4.84,14.32)	8.03 (3.76-11.26)	0.972
Positive GM tests [Cases (%)]	4 (44.44)	3 (37.50)	7 (41.18)	1.000
Types of antibiotics median (IQRs)	5.00 (4.00,8.00)	5.00 (4.00,7.00)	5 (3.5-6.0)	0.894
Combined bacterial infections [Cases (%)]	7 (77.78)	5 (62.50)	12 (70.59)	0.620

Statistically significant intergroup differences (P<0.05) are highlighted in boldface type.

## 4 Discussion

With the global spread of COVID-19, there are more and more reports about patients with combined bacterial and fungal infections, among which fungal infections, especially *Aspergillus* infections, whose higher infection rate and mortality pose a severe threat to the life and health of patients, so it is essential to diagnose the co-infections at an early stage and to make the correct clinical interventions [14]. In our study, the incidence of COVID-19 co-infection with pulmonary mycosis was 29.3%, with a mortality rate of 52.9% (9/17) (**Table 2**). The mortality rate of COVID-19 patients with co-infections with bacterial fungi was as high as 57.14% (4/7). In comparison, the 90-day mortality rate of non-CAPA patients was significantly lower 24.4% (10/41) than that of patients with co-infections with fungal infections (P = 0.035) (**Table 2**). Graciela et al. described the clinical, microbiological, and radiological characteristics of 86 patients diagnosed with probable CAPA in a Mexican hospital. They described the mortality rate, which was as high as 60% in their CAPA patients (Hernández-Silva et al., 2024). In a meta-analysis, the incidence and mortality of CAPA in patients admitted to the ICU were estimated to be 10.2% and 54.9% (Mitaka et al., 2021), and their mortality rate remained high. A meta-analysis by Woon et al. included a total of 729 patients with COVID-19, of whom 14.9% (109/729) were diagnosed with CAPA, with a prevalence ranging from 3.3 to 34.4% between 3.3 and 34.4%, with an all-cause hospitalization mortality rate of 42.6% (OR 3.39; 95% CI,1.97-5.86; P < 0.001) (Chong et al., 2022). It is evident that despite the overall better prognosis of mildly ill patients with Omicron infections, there is a high incidence of CAPA in critically ill patients and an extremely high mortality rate in patients with CAPA.

In treating COVID-19 pneumonia, glucocorticoids, and immunosuppressants have been used as standard therapeutic tools, which play an essential role in controlling the inflammatory response and reducing the mortality of the patients. However, when steroids and immunosuppressants are applied, the risk of CAPA in patients increases. In our study, whether steroids therapy was used or not had no effect on whether CAPA occurred; however, with the prolongation of steroids use (P = 0.002, OR: 1.603 (95% CI: 1.181~2.176)) and the increase in cumulative dose of steroids use ((P < 0.001, OR: 1.012 (95% CI: 1.009~1.015) (**Figure 2**). The incidence of CAPA increased. Hashim et al. concluded in a meta-analysis of 21 studies and 5174 patients that the risk of CAPA was associated with the use of high-dose glucocorticoids and not significantly correlated with low-dose steroids and that patients with COVID-19 were at risk of CAPA even without steroids therapy (Hashim et al., 2023). Gregoire et al. studied 141 patients with CAPA in a single-center retrospective observational study analyzing the incidence and risk factors for COVID-19-associated pulmonary aspergillosis in an intensive care unit. In univariate analyses, patients using steroids had a higher probability of developing CAPA. However, multivariate analyses did not observe an association between steroids and CAPA development (Gregoire et al., 2021). Currently, there are conflicting results in different studies about whether steroids are a risk factor for the development of CAPA, probably because steroids were heavily used as an effective treatment during the COVID-19 period. The baseline conditions of the patients, as well as the dosage of steroids used and the duration of their use, varied in each study center, resulting in conflicting results.

In the present study, we found that the incidence of CAPA increased only when glucocorticoids were used for an extended period. This may be because the steroids were used for a shorter period and did not have a significant immunomodulatory effect on the patients. In our univariate and multivariate analyses of risk factors for CAPA mortality, we found that neither the use of steroids nor the duration of steroids use significantly affected CAPA mortality (**Table 3**). Tolizumab and baricitinib were also commonly used as immunomodulators during the COVID-19 pneumonia epidemic. Several previous studies have identified the use of tocilizumab as a risk factor for CAPA (Cadena et al., 2021; Prattes et al., 2022), and our study found that the use of tocilizumab (P = 0.020) increased the incidence of CAPA. In contrast, the use of baricitinib did not affect the presence of CAPA; in a retrospective study conducted at the National Institute of Respiratory Research (INER) in Mexico City, the incidence of CAPA at its center was only 4.13%. One factor contributing to its low prevalence was the rare use of immunomodulators such as tocilizumab at this center (Hernández-Silva et al., 2024). A meta-analysis showed that the combination of baricitinib with dexamethasone and anti-il-6 inhibitors significantly reduced 28-day mortality (Selvaraj et al., 2022); Albuquerque et al. found that the efficacy of baricitinib was not inferior to that of tocilizumab, and that baricitinib had a shorter half-life compared to tocilizumab, and the shorter half-life of baricitinib compared with tocilizumab was beneficial in reducing the risk of secondary infections (Albuquerque et al., 2023), and the present study found that the use of baricitinib did not increase the prevalence of CAPA as tocilizumab did, and the role that glucocorticosteroids and various types of immunosuppressants and immunomodulators have played in the treatment of COVID-19 pneumonia is well documented, and previous studies have confirmed that glucocorticosteroids may increase the prevalence of COVID-19 pneumonia and that glucocorticosteroids may reduce the risk of secondary infections. Previous studies have also confirmed that glucocorticoids can reduce the mortality rate of COVID-19 ventilated patients (Recovery: Randomised Evaluation of Covid-19 Therapy, 2020), but the side effects should be considered comprehensively, and steroids and immunomodulators can be used in the right amount and at the right time. In addition, we found that patients with diabetes mellitus (P = 0.018), renal insufficiency (P = 0.002), and COPD (P = 0.003) were more prone to CAPA. In the follow-up treatment and monitoring of clinical indicators of patients with COVID-19, we observed an increase in interleukin-6 (P = 0.022), mechanical ventilation (P = 0.002), and an increase in the hospitalization duration (P = 0.021), and increased type of antibiotic use (P = 0.002) were risk factors for the development of CAPA (**Table 2**).

Currently, the incidence of bacterial co-infections in patients with COVID-19 is low, but in the intensive care unit, the incidence of co-infections with bacterial infections and the mortality rate is high due to the prolonged stay in the ICU, the need for prolonged mechanical ventilation, and the high incidence of pulmonary infections in the critically ill patients (Wu H. Y. et al., 2022), and the current reports on both bacterial and fungal co-infections in COVID-19 are extremely rare, and inherently there is no accurate incidence and prognosis of the In our study, we found that the probability of COVID-19 co-infection with both fungal and bacterial infections was about 41.18%, and the mortality rate was as high as 57.14%, and the most common bacterial strain was *Acinetobacter baumannii*, and some previous studies have found that most of the co-infections with bacterial infections of COVID-19 in the ICU were gram-negative (*Pseudomonas aeruginosa*, *Enterobacteriaceae*, and *Klebsiella pneumoniae*) (Bardi et al., 2021; Naseef et al., 2022). Our findings showed that the primary co-infected strain was *Acinetobacter baumannii*. In a retrospective study by Yang et al., a total of 20 critically ill patients with COVID-19, all of whom were admitted to the ICU, were enrolled. The reasons for this may lie in the different ICU environments in different study centers, the use of antibiotics, and the small number of cases preventing a uniform conclusion. All patients with CAPA had large numbers of *Aspergillus* found on more than two sputum cultures or BALF cultures. All patients underwent a serum GM test, which had a positive rate of 41.2% (7/17), and five patients with CAPA underwent an BALF GM test, which was positive in all cases. A previous report found the sensitivity of alveolar lavage fluid GM to be 66.7% in CAPA cases, but only 21.4% were positive for serum GM (Armstrong-James et al., 2020). We can see that BALF is vital in diagnosing CAPA. However, fiberoptic bronchoscopy is rare in many healthcare institutions because the COVID-19 virus can be transmitted by aerosol. CAPA patients with negative serum GM tests are present; some CAPA patients may be underdiagnosed. Therefore, the diagnosis of CAPA should not be limited to serum GM but should be based on sputum culture, CT manifestations, clinical symptoms, and host factors of ECMM/ISHAM.

In this study, the mortality rate of CAPA patients was 57.14% (4/7), which was much higher than the 90-day mortality rate of 24.4% (10/41) in non-CAPA patients (P = 0.004, OR: 0.28, (95% CI: 0.087~0.942)) (**Table 2**), and among CAPA patients, the use of mechanical ventilation (P = 0.040 OR: 10.500, (95% CI: 1.115~98.914)), advanced age (P = 0.043 OR: 1.212, (95% CI: 1.006~1.460)) and significantly elevated CRP levels (P = 0.042 OR: 1.043, (95% CI: 1.002~1.078)) had a poor prognosis (**Figure 3**), and all CAPA patients were treated with antifungal therapy when they were found to be *Aspergillus*, and of the 17 patients with CAPA, nine patients were treated with voriconazole, five patients were treated with esaconazole, and three patients were treated with antifungal therapy with amphotericin B. However, we found that timely antifungal therapy did not significantly improve the patient’s prognosis, which may be related to the patient’s severe condition and prolonged disease duration. For the targets of the drugs as antifungal mainly include: 1. Clinical resolution: ≥72-hour fever remission + respiratory symptom improvement (SOFA score decrease ≥2 points); 2. Microbiological clearance: Negative fungal culture/PCR from respiratory samples on sequential testing; 3. Radiographic stabilization: ≥50% reduction in pulmonary infiltrates on CT within 14 days; 4. Inflammatory normalization: CRP <20 mg/L + IL-6 <40 pg/mL sustained for 5 days (Verweij et al., 2021). The CAPAs mainly occurred in the ICUs. In patients with prolonged disease duration and poor prognosis, their COVID-19 disease duration mainly determines their mortality rate. Antifungal therapy did not improve the prognosis of either proven CAPA, probable CAPA, or possible CAPA (Russo et al., 2024; van Grootveld et al., 2023). Therefore, preventing CAPA is essential in managing critically ill patients with COVID-19.

Our study still needs some improvement. Firstly, this study is a single-center study with a small sample size, so some of the conclusions may differ from those of other centers due to the different diagnostic methods and treatments in each treatment center. Secondly, this study needs more drug resistance monitoring of *Aspergillus* and has limited reference value for using antibiotics to treat COVID-19 combined *Aspergillus* strains. Furthermore, because all patients were COVID-19 critically ill, we excluded patients who died or were automatically discharged within 48 hours of admission. However, these patients may have had a longer course of the disease and fungal infection before admission, so our study found the CAPA incidence and mortality rates. However, they were already at a high level and may be biased compared to the facts. Finally, because critically ill COVID-19 patients are too sick to be diagnosed by histology or direct microscopy, this study was limited to patients with proposed CAPA and suspected CAPA and lacked studies of patients with confirmed CAPA.

## 5 Conclusions

In conclusion, we found a high prevalence of CAPA (29.3%) in cases of severe COVID-19 pneumonia and a mortality rate of 52.9% in patients with CAPA. Risk factors associated with the development of CAPA include underlying diseases such as diabetes, IL-6 elevation, renal insufficiency, and chronic obstructive pulmonary disease, as well as mechanical ventilation, tocilizumab use, and prolonged hospitalization. Previous studies on the use of glucocorticoids have been controversial, with some suggesting that the use of glucocorticoids increases the incidence of CAPA and may worsen the prognosis. In contrast, others have found that using glucocorticoids does not make a significant difference in the occurrence of CAPA and leads to a favorable prognosis. However, our study found that short-term use of glucocorticosteroids in appropriate doses did not increase the incidence of CAPA and helped to improve the prognosis of patients; when glucocorticosteroids were used for a prolonged period, they led to an increase in the incidence of CAPA. Of note, mortality is significantly higher in patients with CAPA compared with non-CAPA patients, and mechanical ventilation, advanced age, and elevated C-reactive protein levels are considered poor prognostic indicators in patients with CAPA. Prevention of CAPA through careful monitoring of high-risk patients, rational use of glucocorticoids and immunomodulators, and effective infection control measures remains a top priority in managing severe COVID-19 cases.

However, we must also note the limitations of our study, including the single-centre design, small sample size, and exclusion of specific patient groups that could have biased our findings. In order to validate our findings and gain further insight into the epidemiology, risk factors, and CAPA management strategies of critically ill COVID-19 patients, future multicenter collaborative studies with larger sample sizes are warranted.

## Data Availability

The original contributions presented in the study are included in the article/supplementary material. Further inquiries can be directed to the corresponding author.
